# A Small-Molecule TrkB/TrkC Ligand Promotes Neurogenesis and Behavioral Recovery Following Traumatic Brain Injury

**DOI:** 10.1089/neur.2024.0117

**Published:** 2025-02-17

**Authors:** Jian Shi, Tao Yang, Yibing Li, Lily Zhong, Frank M. Longo, Stephen M. Massa

**Affiliations:** ^1^ Department of Neurology, San Francisco Veterans Affairs Health Care System and University of California, San Francisco, California, USA.; ^2^ Department of Neurology and Neurological Sciences, Stanford University, Stanford, California, USA.

**Keywords:** cell death, cognitive function, LM22B-10, neurogenesis, traumatic brain injury

## Abstract

Tropomyosin receptor-kinase B (TrkB) and TrkC neurotrophin receptors promote neuronal growth and differentiation during the development and maintenance of structural integrity and plasticity in adult animals. Here, we test the hypotheses that activation of TrkB and TrkC will mitigate neuronal damage and loss, and behavioral deficits induced by traumatic brain injury (TBI). LM22B-10 (C10), a blood–brain barrier permeant small-molecule TrkB/TrkC co-activator, significantly increased proliferation, survival, and enhanced differentiation of neuronal progenitor cells *in vitro*. Following controlled cortical impact injury in rats, LM22B-10 administration increased the proliferation of doublecortin-expressing (DCX) cells in the hippocampus and significantly reduced cell death in the injured cortex. Interestingly, in studies of behavior, LM22B-10 promoted anxiety-like behavior and diminished spatial memory performance in the Barnes maze in sham-TBI animals but improved both of these behaviors in injured rats, a bimodal response suggesting the possibility that excess neurotrophic activity may be detrimental in uninjured animals but compensatory after injury. Thus, TrkB/TrkC agents may constitute a new therapeutic avenue for TBI but will require further study to determine safe and effective applications.

## Introduction

Tropomyosin receptor-kinase B (TrkB) and TrkC signaling play major roles in the development and maintenance of the central nervous system (CNS) and BDNF/TrkB signaling has emerged as a significant factor in a number of pathological CNS processes, including Alzheimer’s and Huntington’s diseases, amyotrophic lateral sclerosis (ALS), and traumatic brain injury (TBI).^1–5^ Though less well studied, NT3/TrkC signaling has been implicated in the regulation of neuronal survival, differentiation, and maintenance in the CNS as well as the peripheral nervous system.^6–9^ NT3 binds to TrkC with high affinity but also binds to the primary nerve growth factor receptor TrkA and TrkB with lower affinity in some cell types.^10,11^, In addition, under some circumstances TrkC activities may oppose those of TrkB, for example, in promoting cell death of axotomized corticospinal neurons in which survival is supported by BDNF.^1,12^ TrkB and TrkC also mediate neurotrophin-driven neurogenesis and neuritogenesis of neural progenitor cells *in vitro* and in the hippocampus.^13–16^

Studies of the effects of activation of Trks on TBI outcomes have yielded variable results, depending at least in part on the methods used to promote receptor activity. Early studies using BDNF protein delivered directly to the brain found no clear effects on post-TBI recovery.^17^ However, positive effects of Trk activation on post-TBI recovery have been observed in studies utilizing genetic methods to upregulate BDNF, as well as with modifications of the protein, and with delivery of small molecule Trk modulators. In a recent study, mice bearing the BDNF-Val66Met mutation, which can interfere with BDNF secretion, exhibited exacerbations of neuronal death, inflammatory responses, and early memory deficits following lateral fluid percussion injury, and injection of an adeno-associated virus (AAV)-BDNF expression vector into the hippocampus and cortex partially mitigated these deficits.^18^ Studies with nanoparticle release of BDNF^19^ and a BDNF-collagen-binding-peptide fusion protein^20^ have reported improvements in behavioral recovery and histological parameters following TBI, further suggesting that distribution and kinetics may be critical factors for pharmacologic neurotrophin use. Another approach using small-molecule Trk receptor activators has also been explored. LM22A-4, a nonpeptide TrkB activator that promotes activation of hippocampal and striatal AKT and ERK1/2 signaling *in vivo*, improved motor learning in rats following direct cortical impact TBI as assessed by progressive improvements in rotarod performance.^21^ In a mouse model of stroke, LM22A-4 was found to promote striatal TrkB phosphorylation, increase subventricular zone and peri-infarct neurogenesis, and improve gait, even when administered 72 h after injury.^22^ Furthermore, another compound reported to activate TrkB, 7,8-dihydroxyflavone (7,8-DHF), has been utilized in several studies of TBI,^23–26^ demonstrating decreases in post-injury cell death and behavioral deficits,^23,24^ protection of dendritic trees and spines, and promotion of neurogenesis.^25,26^

NT-3 and TrkC signaling have been studied in spinal cord injury and brain ischemia models, but less so in TBI. Early studies of NT-3 expression following TBI found that, unlike BDNF, NT-3 was downregulated during the first 24 h after injury.^27,28^ This result, with a report that nestin-conditional NT-3 knockout mice developed smaller infarcts in a stroke model,^29^ led to the proposal that NT-3 might have detrimental effects following injury.^30^ Conversely, protective effects of NT-3 have been reported in other models. The administration of NT-3 following surgical brain trauma decreased brain edema and improved the overall neurological function scores in rats following surgical brain injury, and these effects were reversed with the application of TrkC-siRNA.^31^ Similarly, a study of corticospinal axotomy found that mini-pump administration of NT-3 significantly prevented axotomy-induced death in corticospinal neurons.^30^ Finally, several studies have suggested that delayed administration of NT-3exhibits protective and reparative effects in models of stroke and spinal cord injury^32,33^ and reviewed in Houlten et al.^34^ Thus, NT-3 actions may depend on the mode of delivery and/or the injury model investigated. Moreover, the overall contribution of TrkC signaling remains unclear given the potential for NT-3-mediated TrkB activation.

The blood–brain barrier permeant small-molecule LM22B-10 (C10) activates TrkB as well as TrkC, increasing cell survival, strongly accelerating neurite outgrowth, in some cases exceeding the effects of BDNF.^35^ LM22B-10 also supported substantial early neurite outgrowth in the presence of inhibiting glycoproteins. Examination of the mechanisms of these activities suggested contributions of the activation of both TrkB and TrkC, a requirement for the Trk extracellular domain, and lack of interaction with the p75 neurotrophin receptor (NTR).^35^

In the present study, we investigated the effects of LM22B-10 on neuronal survival and neural progenitor proliferation and differentiation *in vitro* and *in vivo*, and on behavioral deficits following injury. To test these, we utilized cell lines and primary cell cultures, and evaluated histological and behavioral outcomes in rats after TBI. The results indicate that small molecule-mediated modulation of TrkB and TrkC activity may reduce both early and later sequelae of the post-TBI injury process but may also induce behavioral deficits in normal animals.

## Materials and Methods

### Controlled cortical impact injury, LM22B-10, and 5′-bromo-2′-deoxyuridine administration

All animal protocols were approved by the Institutional Animal Care and Research Advisory Committee at the San Francisco VA Medical Center and by the Association for Assessment and Accreditation of Laboratory Animal Care. For all injury experiments, 5- to 6-week-old male Sprague-Dawley (SD) rats were purchased from Charles River Lab. Animals were 7- or 8-week-old when TBI was performed as described by Shi et al.^36^ Briefly, moderate cortical injury was induced left of the midsagittal suture centering at AR 0.0 mm; ML: 3.0 mm, using a custom impactor with a 2.5 mm rounded tip, penetrating at a velocity of 1.5 m/sec to a depth of 2.5 mm with a dwell time of 120 ms. Sham animals received the same anesthetic injection and incision but did not undergo craniotomy and impact. LM22B-10 (2-[[4-[[4-[Bis-(2-hydroxyethyl)-amino]-phenyl]-(4-chloro-phenyl)-methyl]-phenyl]-(2-hydroxy-ethyl)-amino]-ethanol) obtained from Ricerca Biosciences LLC (Concord, OH), was prepared for delivery in Cremophor/Phosphate-buffered saline (PBS) following the protocol of Yang et al.^35^ and 10 mg/kg LM22B-10 or vehicle alone was injected intraperitoneally (IP).

**Fig. 1.**
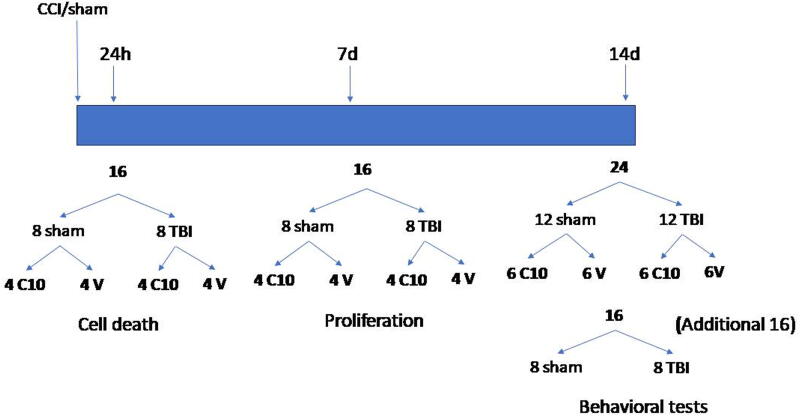
Animal groups and treatment times.

For the cell death study, four groups of four rats, each receiving controlled cortical impact (CCI), were treated with LM22B-10 or vehicle once daily beginning immediately following injury, and four rats in each group were sacrificed at 1 day after injury for histological analysis. For the proliferation study, four groups of four animals were treated with LM22B-10/vehicle, along with 50 mg/kg (∼200 μL) 5′-bromo-2′-deoxyuridine (BrdU) IP once daily for 7 days before sacrifice. The first injection of BrdU was immediately after TBI, and the last injection was performed 24 h before euthanasia. A third cohort with four groups of six animals each was treated with LM22B-10/vehicle for 14 days and sacrificed following behavioral testing. Finally, an additional two groups of eight animals treated with vehicle, eight sham, and eight TBI were included in open-field testing, and those animals were treated by the same vehicle after the same levels of TBI and sham procedures. The figure 1 shows all animal numbers and groups, C10/vehicle injection periods, and study objectives.

## Viability, Proliferation, and Differentiation of Cultured Cells and BrdU Detection

P19 mouse embryonic carcinoma cells, used for dose–response studies, were purchased from the American Type Culture Collection (ATCC) and grown in a minimal essential medium (Invitrogen), supplemented with 5% heat-inactivated fetal calf serum in 5% CO_2_ at 37°C.^37^ Cell viability was measured using the tetrazolium-based Cell Counting Kit-8 (Dojindo Molecular Technologies, Inc., Rockville, MD), as previously described.^36^

Adult hippocampal neural progenitor cells (AHP) were isolated and cultured as described in Shi et al.^36,38^ from the hippocampi of 5-week-old female SD rats. Human embryonic neural stem (ENS) cells were purchased from Chemicon (St. Louis, MO) and cultured in ENStem-A medium containing 10 ng/mL bFGF-2. These cells express high levels of the neural stem cell markers Nestin and Sox 2, express Oct-4 at low levels, and can differentiate into multiple neuronal phenotypes (protocols detailed by the manufacturer [Cat. SCM 017, Chemicon]). For treatments *in vitro*, LM22B-10 was dissolved in 0.04 N HCl in water and diluted in a culture medium to a final dilution of 1:10,000 HCl. For vehicle controls, HCl was diluted in culture media to the same concentration. Cell proliferation was measured using BrdU incorporation with BrdU immunostaining as in Yang et al.^35,36^ For cultured cells, BrdU (1 µM) was added with vehicle or LM22B-10 for 48 h before fixation and staining. Using Image J software, the number of BrdU-incorporating cells was counted in each of 5–10 random images for each treatment condition from each of the three independent experiments. For differentiation assays, cultures of AHP and ENS were maintained in a differentiation medium with withdrawal of FGF for 48 h and exposure to BDNF, LM22A-10, or vehicle alone, then fixed with 4% paraformaldehyde (PFA), and stained with anti-β-tubulin III (Tuj1) antibody, which allowed visualization of neurites and cell bodies. Using Neurolucida software (v.10, MBF Biosciences), neurite length was measured for each Tuj1 positive cell as well as the number of positive cells. Random images, 5–10 random images for each condition from three independent experiments, were collected and measured as previously described.^35^

### Immunofluorescence and Fluoro-Jade C staining

Animals deeply anesthetized with ketamine and xylazine were perfused with 200 mL PBS followed by 200 mL 4% PFA; brains were dissected and embedded in optimal cutting temperature (Tissue-Tek, Sakura, Torrance, CA), and 20 µm cryotome sections were mounted on Super-Frost Plus slides (Fisher Scientific, Waltham, MA) and stored at −20°C.

For Fluoro-Jade C (FJC) staining, sections were dried at 60°C for 1 h and then stained following the manufacturer’s protocol (Cat. AABH9A22D7AF, Sigma) with modifications specified in Shi et al.^36^ The ruler of confocal microscope (Zeiss LSM 510) was used to find the location as shown in Figure 4A, and the machine automatically scanned 2 × 2 areas (4 × 450 µm^2^) at 40×. For BrdU staining, sheep anti-BrdU 1:600 (Cat. Ab1893, Abcam, Cambridge, MA) was used, and sections were soaked in 50% formamide/2 × saline-sodium citrate (SSC) (0.3 M NaCl, 0.03M sodium citrate) for 2 h at 65°C, washed for 5 min in 2× SSC at room temperature (RT), incubated for 30 min in 2 N HCl at 37°C, and was held for 10 min in 0.1 M boric acid (pH 8.5) at RT.^36^

For immunofluorescence, after several rinses in tris buffered saline with Tween 20 (TBST) (0.15 M NaCl, 0.1 M Tris-HCl, 0.1% Triton X-100, pH 7.5), sections were incubated in TBST/3% normal donkey serum for 30 min and incubated overnight with primary antibodies. Primary antibodies included: rabbit anti-doublecortin-expressing (DCX) 1:500 (Cat. 48-1200, Invitrogen); mouse anti-β-tubulin III 1:1000 (Cat. G7121, Promega); anti-TrkB polyclonal antibody 1:1000 (Cat. PA5-78405, Invitrogen); rabbit anti-TrkC monoclonal antibody 1:500 (Cat. 3376, Cell Signaling Technology, Beverly, MA); and sheep anti-BrdU 1:600 (Cat. Ab1893, Abcam, Cambridge, MA). Secondary antibodies (Invitrogen Life Technologies, Grand Island, NY) were used at concentrations of 1:300, and DAPI (1:1000; 4′,6-diamidino-2-phenylindole-2 HCl) was utilized to stain nuclei. Slides were cover-slipped with Immunomount (Shandon, Pittsburgh, PA), and stored at 4°C.

### Cell counting

Immunofluorescent cells in the dentate gyrus and cortex were counted in every fifth section throughout the injured area of each animal. Note that 40× images of immunohistochemical sections of the entire dentate gyrus of all groups of animals were obtained using a Zeiss 510 Confocal microscope, utilizing the microscope’s tile scan mode, and assembled using Photoshop. *In vitro* cell counts were performed on at least three samples per group. Five randomly selected fields in the center of each cover-slip were imaged and counted by an observer blinded to the treatment group.

For evaluation of cell injury following TBI, FJC-positive cells were counted in the lesioned and non-lesioned cortex in five coronal sections spaced 100 µm apart and centered on the lesion epicenter or the equivalent location on the contralateral side. Four (2 × 2) 450 µm^2^ areas were photographed for later analysis: an area centered 200 µm lateral to the lesion edge and midway through the cortical depth.

For differentiated cells, neuronal complexity was measured using the complexity score calculated in Neurolucida. Neurolucida complexity scores were defined as:

Scores = (∑ending orders + #endings) * (∑dendritic length/# primary dendrites), where ending order = number of tributaries encountered tracing from ending to cell body, per neurite, and using Sholl analysis as previously described.^39^

## Barnes Maze

Learning and memory behaviors were assessed using the Barnes maze (BM) test.^40^ One week following treatments, animals were evaluated by an examiner naive to treatment. Animals were trained to locate an escape hole for 5 days in two daily sessions 3 h apart. Time to reach the hidden hole (latency), path length, and walking speed were recorded with a Noldus Instruments EthoVision video tracking system (Noldus Information Technology, Sterling, VA).

## Open Field and Elevated Plus Maze

Overall activity and anxiety-like behavior in rodents were assessed using open-field testing.^41^ Animals were placed in a brightly lit square plexiglass enclosure (40 × 40 inches), surrounded by infrared photocells (Hamilton & Kinder, San Diego, CA, USA), and their spontaneous movements were recorded. For analysis, the arena was divided into zones consisting of a center region (15 × 15 inches), four corner regions of 7.5 × 7.5 inches each, and a peripheral region (the remaining area). Animals were allowed to explore for 10 min, during which the total distance traveled and inner/outer zone entries were recorded as previously described.^42,43^ After each rat was tested, the enclosure was cleaned with 30% ethanol to reduce its residual odor.

Anxiety-like behavior was further evaluated using an elevated plus maze (EPM) consisting of two opposite open and bright arms and two opposite closed and dim arms (45 cm × 15 cm),^44^ each connected to a central area (15 cm × 15 cm), elevated 70 cm above the ground. Animal movements are tracked using EthoVision system video tracking. Normal animals explore the novel environment avoiding open, elevated spaces in favor of enclosed spaces. Changes in this behavior, such as significant reductions in open and bright areas, have been used as a measure of anxiety in rats.^45^ Animals were placed into the central area under dim lighting and their behaviors were observed and recorded for 5 min. After each rat was tested, the arms and central area were cleaned with 30% ethanol.

### Statistical analyses

Statistical analyses were performed using SigmaPlot 14 or Prism 8.0.4. Normality testing (Shapiro–Wilk) was performed before analysis and parametric or nonparametric tests were chosen based on those results. Student’s *t* test was employed for parametric group comparisons, and for nonparametric analyses, the Mann–Whitney U test was used. BM data were rank-transformed prior to repeated measures ANOVA with Holm–Sidak *post hoc* testing. *p* Values of <0.05 were considered significant. Graph bars indicate mean + standard error throughout the figures.

## Results

### LM22B-10 promotes the proliferation differentiation, and survival of neural progenitors

**FIG. 2.**
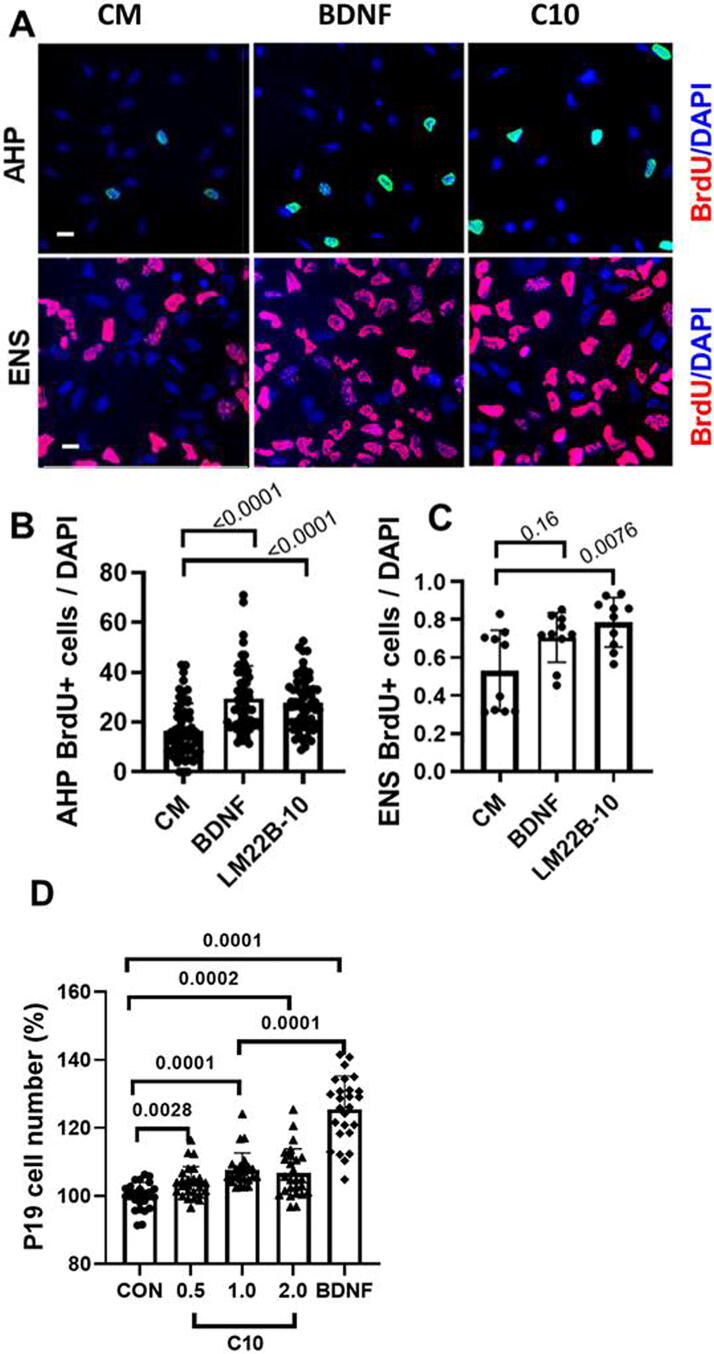
Neurotrophic agents promote neural progenitor cell proliferation and survival *in vitro*. **(A)** Representative images of adult hippocampal progenitor (AHP) and embryonic neural stem (ENS) cells in culture media (CM) containing 0.1% FBS in the presence of the 0.7 nM BDNF or 1 µM LM22B-10. Cultures were maintained for 48 h prior to staining for BrdU; quantitation of BrdU+ cells. **(B)** AHP cells; LM22B-10 and BDNF increased BrdU+ cells, *p* < 0.0001 vs. CM, *n* = 20–40. **(C)** ENS cells; LM22B-10 increased BrdU+ cells, *p* < 0.0076 vs. CM, *n* = 10–20, while changes with BDNF were not significant. **(D)** P19 cell counts; LM22B-10 at 0.5, 1, and 2 µM similarly increased cell numbers, *p* = 0.0028, 0.0001, and 0.0002 vs. CM, *n* = 20. BDNF increased cell numbers, *p* = 0.0001 vs. CM, *n* = 20, to a greater extent than observed in 1 µM LM22B-10-exposed cells, *p* = 0.0001, *n* = 20 for the number of samples and using Student’s *t* test. In these studies, *n* represents sample numbers, the statistical significance of individual comparisons utilizes Student’s *t* test unless otherwise stated, and scale bars represent 10 µm. BrdU, 5′-bromo-2′-deoxyuridine.

The roles of TrkB and TrkC in the proliferation and differentiation of neural progenitors are complex, with activation of TrkB and TrkC reported to promote progenitor survival and proliferative capacity,^46,47^ while *in vivo* effects on proliferation may be dominant with little effect on survival, unlike *in vitro* results.^48^ Moreover, conversely, in a recent study, blocking TrkB activation was found to increase the proliferation of a subpopulation of neural progenitor cells in the hippocampus of mice.^49^ Given these findings, it was of interest to determine the effects of LM22B-10 on progenitor cell populations. AHP and ENS cells were treated with BDNF (0.7 nM), LM22B-10 (1 µM), or control medium (CM) for 48 h and BrdU uptake was assessed as a measure of cell proliferation. Both BDNF and LM22B-10 increased the proliferation of AHP cells to similar degrees 57.4 ± 8.5% and 73.5 ± 6%, each *p* < 0.0001 versus CM, *n* = 20 (Fig. 2A, 2B). In contrast, LM22B-10 increased the proliferation of ENS cells by 48.3 ± 3%, *p* = 0.0076 versus CM, *n* = 10, while the increase due to BDNF did not reach significance (*p* = 0.16) (Fig. 2A, 2C). Similar responses to the agents were found in P19 cells, which showed increases with 0.5–2 µM LM22B-10, though in contrast to the pattern in ENS cells, these responses were significantly less than those seen with BDNF (Fig. 2D).

**FIG. 3.**
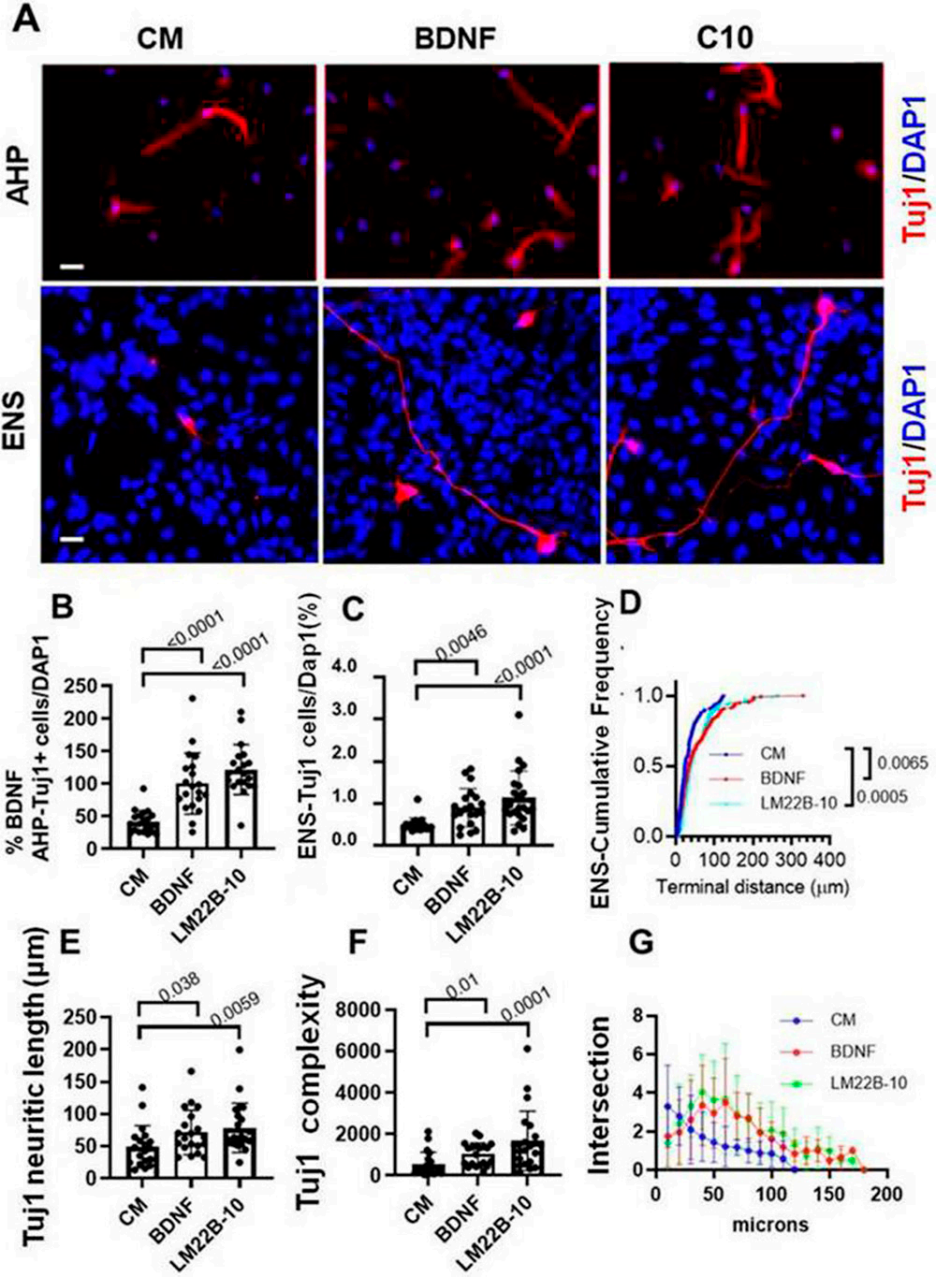
LM22B-10 and BDNF promote neuronal differentiation of progenitors *in vitro*. **(A)** Representative images of AHP and ENS cells in differentiation CM in the presence of 0.7 nM BDNF or 1 µM LM22B-10. Cultures were maintained for 48 h before staining for Tuj1-expressing neurons with DAPI for nuclei. **(B)** Quantitation of Tuj1+ cells, LM22B-10 or BDNF vs. CM *p* < 0.0001, *n* = 20. **(C)** Tuj1+ ENS cell counts, BDNF vs. CM *p* = 0.0046, *n* = 20, LM22B-10 vs. CM *p* < 0.0001, *n* = 20. **(D)** ENS neurite length cumulative frequency distributions; both agents shifted neurite length distributions toward longer lengths, increasing under each culture condition obtained from three independent experiments, *p* = 0.0065 BDNF vs. CM and *n* = 10, *p* = 0.0005 C10 vs. CM and *n* = 10. **(E)** LM22B-10 and BDNF increased the mean length of T+ ENS cells, BDNF vs. CM, *p* = 0.036 and *n* = 20, C10 vs. CM, *p* = 0.0058 and *n* = 20. **(F)** ENS cell neuritic complexity; LM22B-10 and BDNF increased neurite complexity (as described in *Methods*), BDNF vs. CM, *p* = 0.01, *n* = 20, C10 vs. CM, *p* = 0.0001, *n* = 20. **(G)** Intersection distances in Sholl analysis; BDNF and LM22B-10 produce similar distributions of neurite lengths in ENS T+ cells. In this study, scale bars represent 10 µm. AHP, adult hippocampal progenitor; ENS, embryonic neural stem; CM, culture media.

Trk activation may also induce differentiative programs in progenitor cells and developing neurons, including promoting neurogenesis and the elaboration of neurites.^50,51^ To examine LM22B-10 effects on neurite outgrowth and generation of neurons, AHP and ENS cells were treated for 48 h with BDNF or LM22B-10, and numbers of Tuj1 expressing neurons were determined in AHP cultures (Fig. 3A, upper panels, 3B), as well the length of elaborated neurons was determined in ENS cultures (Fig. 3A, lower panels, 3C–F). BDNF increased 142 ± 17.8% the percentage of Tuj1-expressing cells in AHP cultures relative to CM alone (*p* < 0.0001, *n* = 10), with an increased 193 ± 15% response to LM22B-10 (*p* < 0.0001, *n* = 10; Fig. 3B). Both agents shifted neurite length distributions toward longer lengths in ENS cells (Fig. 3D), increasing the average length (BDNF *p* < 0.0065 vs. CM, *n* = 20 and LM22B-10, *p* < 0.005, *n* = 20, Fig. 3E), and also increased complexity as reflected by Neurolucida complexity scores (BDNF vs. CM, *p* = 0.01, *n* = 20, LM22B-10, *p* = 0.001, *n* = 20, Fig. 3F) and intersection distances in Sholl analysis (Fig. 3G). Thus, in addition to prior reported effects on the survival of primary neuronal cells,^35^ the compound may have differential effects on the proliferation and differentiation of neural progenitors *in vitro*.

### LM22B-10 decreases neuronal death following TBI

Given the effects of LM22B-10 on progenitor and more mature neuronal cells, it was of interest to determine whether it might similarly improve cell survival and numbers *in vivo* following injury, potentially providing substrates for functional recovery.

**FIG. 4.**
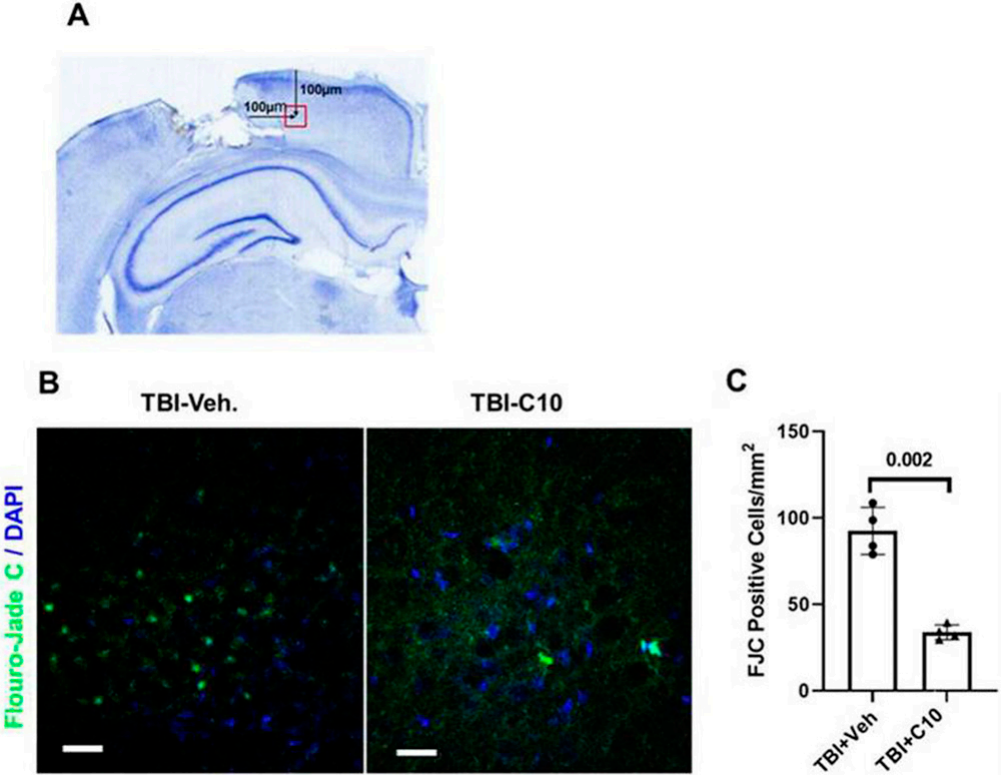
LM22B-10 mitigates neuronal injury following TBI. Fluoro-Jade C (FJC) staining 24 h following TBI in regions of cortical injury was assessed as described in the *Methods.* **(A)** Representative showing localization of the measured area in each brain section. **(B)** Representative images of FJC-positive cells in the area measured. **(C)** Quantitation of FJC-positive cells; LM22B-10 treatment reduced FJC-positive cells compared with vehicle-treated animals, C10 vs. vehicle (Veh), *p* = 0.002, *n* = 4 for induvial animal of all groups. Each scale bar in the figures represents 100 µm. TBI, traumatic brain injury.

The CCI produced local cortical neuronal cell death, as indicated by FJC staining 24 h post-injury. FJC-positive cells were observed in the ipsilateral cortex, and the specific area we observed is shown in Figure 4A. The location was found in each slice using a ruler of confocal as shown in the figure to prevent errors caused by different injured sites selected. Intraperitoneal LM22B-10 administration following injury resulted in a 58.6 ± 8.9% reduction in FJC-positive cells in the injured cortex compared with vehicle-treated injured cortex (Fig. 4B and 4C). This reduction in cell death is similar to that mediated by the p75NTR modulator LM11A-31 observed in this model.^36^

### LM22B-10 stimulates the proliferation of adult hippocampal DCX progenitor cells

TrkB has previously been found to be expressed throughout the stages of neuronal maturation in the hippocampus including in immature DCX+ neurons^50^; in contrast, TrkC has been reported to be expressed in neural stem cells, but its presence at early committed neuronal stages has not been described. Since LM22B-10 may activate TrkB and TrkC,^35^ and increased cell viability of p19 cells as well as the proliferation and neuronal differentiation of AHP and ENS cells, we hypothesized the compound might increase proliferation, survival, and/or differentiation of neural progenitor cells in the hippocampus.

**FIG. 5.**
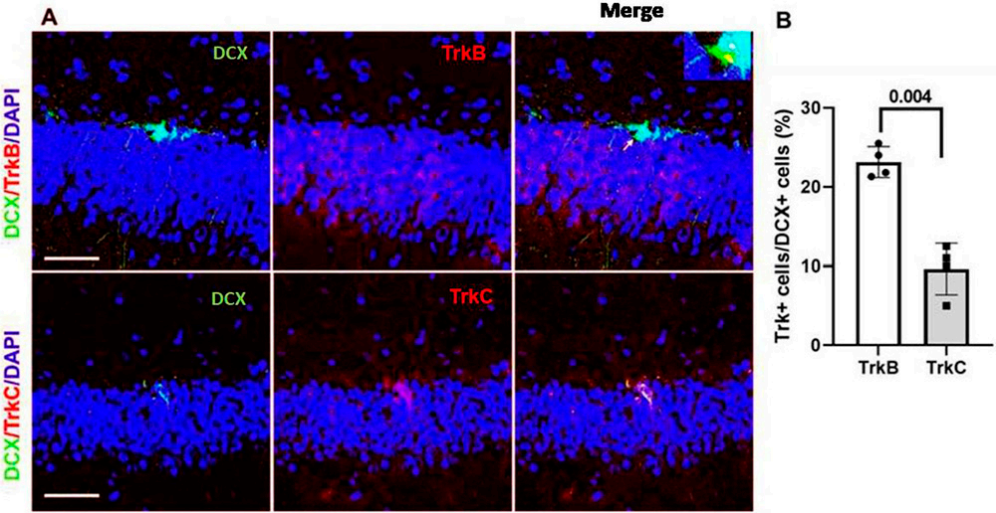
TrkB and TrkC expression in hippocampal DCX+ cells. **(A)** Representative images of DCX+/TrkB+ (upper panel) and DCX+/TrkC+ (lower panel) cells in sham-TBI hippocampal dentate gyri. **(B)** Quantitation of DCX+/TrkB+ relative to total DCX+ cells showed that these were approximately twice as abundant as DCX+/TrkC+ cells in the hippocampus, *p* = 0.004, *n* = 4 for individual rats of all groups. Each scale bar in the figures represents 100 µm. TrkB, tropomyosin receptor-kinase B; DCX, doublecortin-expressing; TBI, traumatic brain injury.

**FIG. 6.**
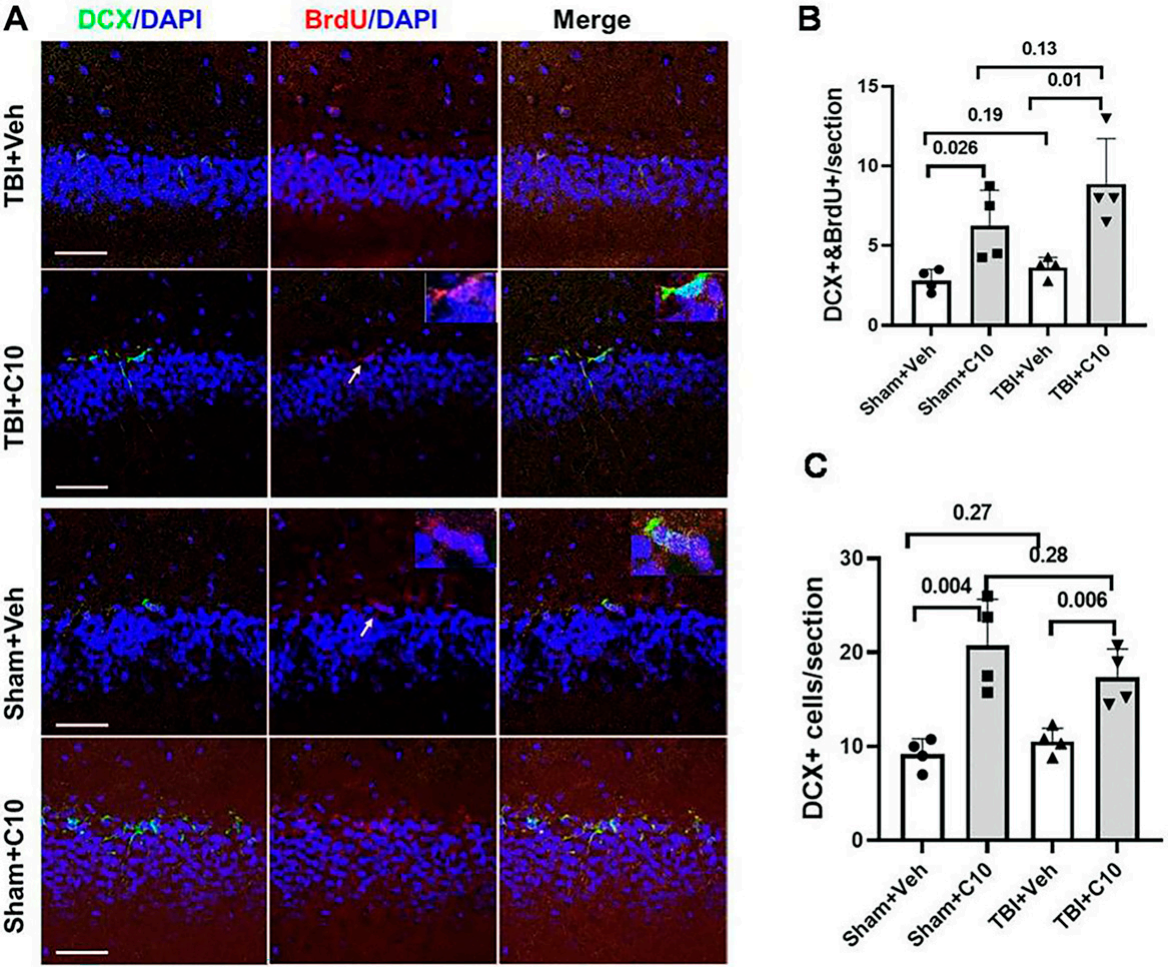
LM22B-10 increases neural progenitor differentiation in the hippocampus following TBI. **(A)** Representative images of hippocampal dentate gyri stained for DCX and BrdU 7 days following TBI or sham surgery; row 1, TBI+Veh; row 2, TBI+LM22B-10; row 3, Sham+Veh; row 4, Sham+LM22B-10. **(B)** Quantitation of DCX+/BrdU+ cells 7 days following TBI; Sham+Veh vs. Sham-C10, *p* = 0.026, TBI+Veh vs. TBI+C10, *p* = 0.01, with other comparisons nonsignificant as indicated, *n* = 4 for all groups. **(C)** Quantitation of DCX+ cells, Sham+Veh vs. Sham+C10, *p* = 0.004, TBI+Veh vs. TBI+C10, *p* = 0.006, with other comparisons nonsignificant as indicated, *n* = 4 for individual animals in each treatment group. Each scale bar in the figures represents 100 µm. BrdU, 5′-bromo-2′-deoxyuridine; TBI, traumatic brain injury; DCX, doublecortin-expressing.

Investigation of NTR expression in immature neurons showed that 22 ± 6.2% of the DCX+ cells in the hippocampal subgranular zone (SGZ) co-expressed TrkB, and fewer, 9.8 ± 5.8% of DCX+ cells, expressed TrkC (Fig. 5A, 5B). These results suggest that LM22B-10 acts through receptors for either TrkB or TrkC, or both, to enhance neuroregeneration after TBI. In vehicle-treated animals undergoing TBI, DCX expression appeared similar to that of sham levels, at 7 days following surgery (Fig. 6A, first column, first and third rows; 6B). The number of DCX-positive cells in the dentate also exhibited nuclear BrdU incorporation, a reflection of recent proliferative activity. Numbers of DCX+/BrdU+ cells in vehicle-treated TBI animals were also similar to those in sham animals (Fig. 6A, third column, 6C). Interestingly, in LM22B-10-treated animals, the numbers of DCX+ cells were increased by approximately 65.4 ± 7.2% compared with vehicle treatment in TBI animals (Fig. 6A, first column, second and fourth rows, 6B). Similarly, DCX+/BrdU+ cells were increased by 106 ± 4.8% compared with vehicle-treated TBI animals in both sham and TBI groups. This finding suggests that C10 may promote a significant augmentation of cells in earlier stages of neurogenesis independent of the injury state.

### LM22B-10 improves cognitive performance and modulates anxiety-like behaviors following TBI

**FIG. 7.**
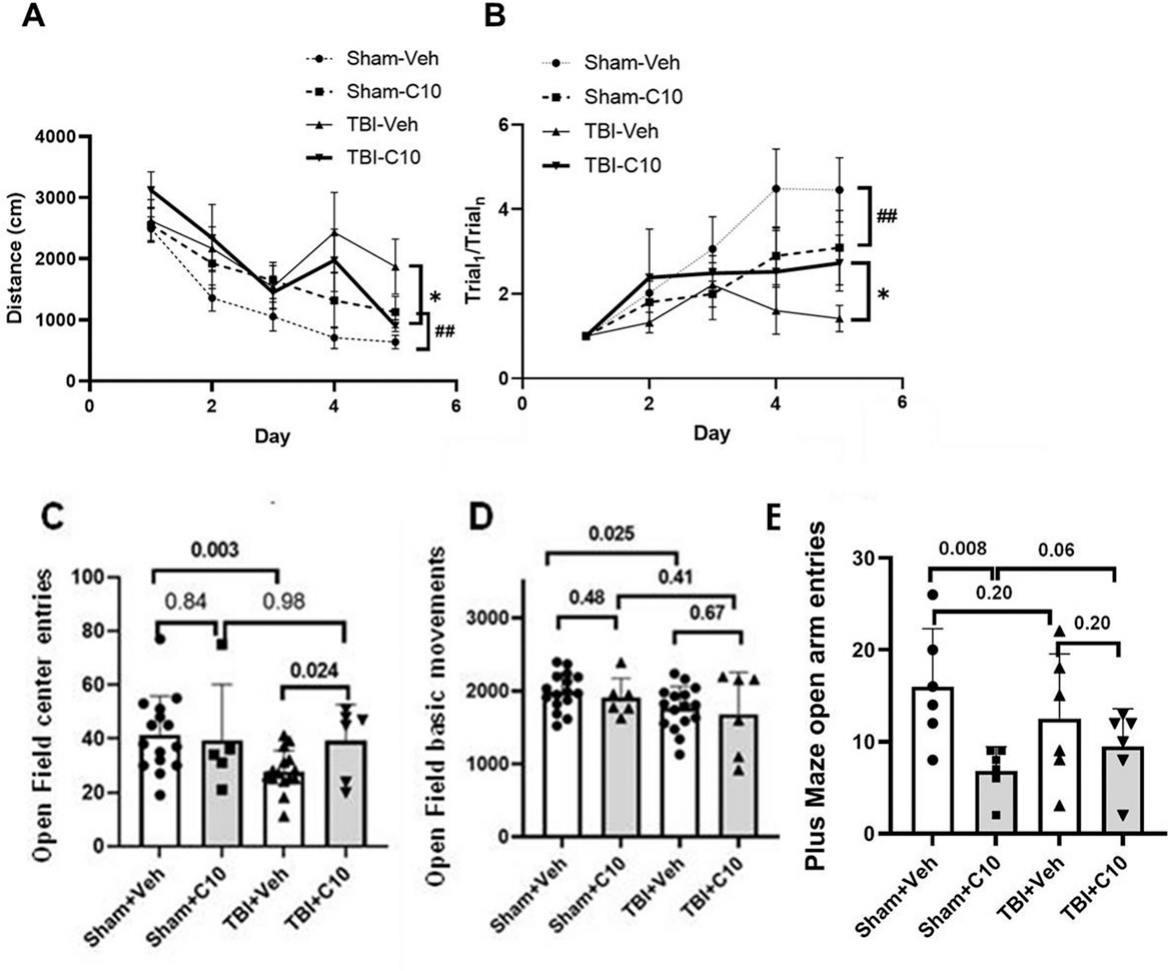
LM22B-10 modulates cognitive deficits and experimental stress following TBI. **(A)** Distances traveled in the Barnes maze (cm). For TBI animals, the distance between TBI-C10 and TBI-Veh was significantly changed, *p < 0.001, *F* = 4.186, and *n* = 6 (by repeated measures ANOVA); for sham animals, the distance between Sham-C10 and Sham-Veh was also significantly changed, ##*p* < 0.01, *F* = 4.564, and *n* = 6 (by repeated measures ANOVA). **(B)** For learning rate, it was calculated as first trial divided by subsequent trial distance for each animal. TBI caused a significant delay in task acquisition which was partially reversed by treatment with LM22B-10; *p < 0.05 TBI-C10 vs. TBI-Veh, *n* = 6 by repeated measures ANOVA. For sham animals, LM22B-10 caused a learning rate decrease in the Barnes maze, ##*p* < 0.01 Sham-C10 vs. Sham-Veh, *n* = 6 by repeated measures ANOVA. **(C)** Center entries in open-field testing. TBI was associated with significantly fewer entries into the center area, Sham+Veh vs. TBI+Veh, *p* = 0.003, *n* = 14; LM22B-10 treatment normalized this parameter, TBI+C10 (*n* = 6) vs. TBI+Veh (*n* = 14), *p* = 0.024. **(D)** Basic movement in the open field; TBI decreased the overall movement (*p* = 0.025, Sham+Veh vs. TBI+Veh, *n* = 14), but there was no significant effect of LM22B-10 on sham or TBI outcomes. **(E)** In the elevated plus maze (EPM), the LM22B-10 treatment significantly reduced the number of entries by sham-surgery animals compared with the Sham+Veh group (*p* = 0.008, *n* = 6). However, there was a trend toward fewer entries in the TBI group compared with the Sham+Veh group, although it did not reach significance (*p* = 0.2, *n* = 6). LM22B-10 treatment resulted in an increase in entries for TBI animals (Sham+C10 vs. TBI+C10, *p* = 0.06, *n* = 6 [Mann–Whitney U]). However, there was no significant difference between the TBI+Veh and TBI+C10 groups. TBI, traumatic brain injury.

Given the potential mitigating effects of LM22B-10 on CCI-induced neural cellular responses, including early cell death, we hypothesized that the compound might counteract injury-induced deficits in spatial learning. To examine the effects of LM22B-10 on spatial memory and motor function following TBI, BM performance^41^ and open-field locomotion were assessed after 2 weeks of continuous treatment. In BM testing, there were no significant differences in average walking speeds between the different treatment groups of rats during visible platform sessions. Sham-TBI animals given LM22B-10 once daily for 2 weeks exhibited a small but significant decrease, as confirmed by repeated ANOVA analysis of 5-day learning processes, in learning rate in the BM relative to animals given vehicle (Fig. 7A). This subtle decrease was further evident from a slightly reduced slope in the early portion of the corresponding learning curve (Fig. 7B), indicating a potential alteration in their learning behavior, which could be associated with factors such as cognitive processing efficiency. In contrast, following TBI, learning and memory deficits were partially recovered in TBI-exposed animals given LM22B-10 compared with those given vehicle (Fig. 7A and 7B). Thus, while LM22B-10 demonstrated the capacity to ameliorate cognitive deficits induced by TBI, it also appeared to exert a subtle influence on cognitive behavior in uninjured animals. Overall, these findings suggest that LM22B-10 exhibits both modulatory effects on cognitive function.

Since we observed significant differences in learning and memory behavioral tests between sham animals treated with vehicle and C10, we decided to investigate anxiety-like behavior in order to elucidate this phenotype. To accomplish this, we utilized the open-field and EPM tests. In order to increase the statistical reliability and feasibility of the open-field test, we combined the two groups of vehicle-treated animals for this study. Notably, there were no differences in overall locomotor activity in the open-field test following drug treatment, although slight differences in basal locomotion were observed between sham- and TBI vehicle-treated animals (Fig. 7D), indicating that the drug had no overall effect on locomotor behavior. In the open-field test, exposure to the center of the field is considered a stress-inducing stimulus that elicits anxiety-like behavior, which is indicated by a decrease in central entries.^52^ LM22B-10 did not alter the number of center entries in sham animals (Fig. 7C). However, following TBI, animals treated with vehicle exhibited a significant reduction in center entries, which was restored to normal levels with LM22B-10 treatment (Fig. 7C), indicating a decrease in anxiety-like behavior. There were no significant changes in other open-field parameters, such as distance traveled in the center field and time spent in the center field.

In the EPM test, reduced entry into the open/bright arms is considered to reflect elevated levels of anxiety or stress in rodents. LM22B-10 treatment of sham animals resulted in significant changes in behavior, with a decrease in the frequency of entry into the open/bright arms of the maze (Fig. 7E). TBI also led to a decrease in entries compared with sham animals. In TBI-exposed animals treated with LM22B-10, there was a significant increase in entries compared with sham animals treated with the compound, indicating distinct effects due to the injury. Notably, unlike the results of the open-field test, there was no significant recovery of performance between the TBI+Veh and TBI+C10 groups in this EPM test.

## Discussion

Previous studies with LM22B-10 found that it enhanced hippocampal neuron neurite outgrowth and survival *in vitro* utilizing both TrkB and TrkC receptors, and not TrkA or p75NTR.^35^ In this study we find that LM22B-10 also has broad effects on neural progenitor(-like) cells, increasing cell proliferation and differentiation *in vitro*. TrkB, with its cognate ligand BDNF, has been reported to promote hippocampal progenitor cell proliferation, though without an effect on survival, at least *in vivo*.^50^ BDNF/TrkB signaling also promotes differentiation and maturation of later stage progenitors.^53^ The role of TrkC in neural progenitor proliferation and survival is less well studied, though in one recent study, downregulation of TrkC was associated with decreased proliferation of opossum cortical neurons^48^; however, work on its canonical ligand NT-3 has suggested a principal effect on differentiation without clear effects on proliferation or survival.^16,54^ This work is complicated by the partial activation of TrkB by NT-3,^35^ and the potential for receptor co-expression in some cell types.^55^ Since initiation of differentiation is associated with cessation of proliferation and many cells entering this path will undergo apoptosis, it remains for further study whether the effects of LM22B-10 are dominated by inhibition of cell death or whether there is a significant promotion of differentiative signaling, and which effects may depend on TrkB, TrkC, or both together. In addition, the lack of interaction with p75NTR may play a role, and possible interactions with truncated receptor forms remain to be investigated.

*In vivo* and following TBI, the effects of LM22B-10 treatment show several similarities as well as potentially important differences with those of other pharmacologic modulators of neurotrophin signaling. *In vivo* following TBI, LM22B-10 substantially decreases neuronal cell death in the area surrounding the injury, similar to that seen in a study of 7,8-DHF in mice^24,56^ and in a study of the p75NTR modulator LM11A-31.^36^ Differences appear in the examination of progenitor cell effects. In a study of 7,8-DHF effects in mice, hippocampal dentate DCX+ cells were found to increase with compound treatment following TBI but not in animals receiving the sham procedure. In addition, the compound did not appear to enhance proliferation as indicated by BrdU incorporation.^25^ In contrast, in the present study, LM22B-10 increased DCX+ cells to a similar extent in both TBI-exposed and sham animals and was associated with an increase in BrdU incorporation, consistent with proliferation in the progenitor cell population. Thus, the *in vivo* response of progenitors to LM22B-10 mirrors the findings *in vitro*. Since a proportion of these cells express one or both of TrkB and TrkC, the response to the coactivation of these receptors might contribute to the observed differences in proliferation relative to singular TrkB activation. However, again, differences between the models and relative dosages used might also play significant roles. Determination of the relative contributions of these factors will require further study.

BDNF/TrkB signaling is well known to be involved in several aspects of behavior, including hippocampal learning and memory (reviewed in Tyler et al.^57^) and mood.^58,59^ One potential significant mechanism through which neurotrophic signaling might influence both memory and mood is through modulation of hippocampal neurogenesis, which has been extensively linked to aspects of memory formation, depression, and other behaviors (reviewed in Anacker et al.^60^). In the present study, LM22B-10 appeared to slightly but significantly decrease performance in the BM in the uninjured state and increased learning following TBI. Since there is an apparent increase in neurogenesis (DCX+ cells) associated with LM22B-10 administration in both populations, these findings suggest that enhanced neurogenesis may be detrimental under normal physiological conditions, and/or that hippocampal neurogenesis is just one of several potential contributors to performance in this test that are affected by LM22B-10. The latter is consistent with a study from Sahay et al.^61^ who found that increased neurogenesis in mice increased animal ability to discriminate between the contexts of stimuli, but had no effect on their spatial learning, novelty recognition, or fear conditioning, each of which has potential roles in the BM. Interestingly, the BM results are reflected to some extent in the results of plus maze testing, with LM22B-10 administration associated with decreased function in uninjured animals, and with relative increases following TBI. Thus, several mechanisms and anatomical substrates may contribute to the responses observed which remain to be determined. Furthermore, the small decrease in learning and a larger increase in anxiety-like behavior of sham-surgery animals receiving the compound may indicate that whatever compensatory mechanisms the compound stimulates, they may be detrimental to normal physiological functions.

Limitations of the study include: (1) dose timing; the immediate administration of the compound after TBI may reduce relevance to the many clinical situations where treatment delays are common. Further studies will be required to determine the timing of administration of LM22B-10 similar compounds for optimal effectiveness and safety. (2) The unexpected effects observed in sham animals require replication for further characterization and validation, and (3) the current study’s approach to allometric scaling and dosing in animals may not fully capture the optimal therapeutic window or the pharmacological mechanisms behind the observed adverse effects. Further investigation is necessary to determine the most effective and safe dosing strategies and to understand the underlying pharmacological pathways. Addressing these limitations is crucial for improving the clinical relevance and applicability of our findings.

## Abbreviations Used

AHP: Adult hippocampal neural progenitor cells
ALS: amyotrophic lateral sclerosis
ATCC: American Type Culture Collection
CCI: controlled cortical impact
CNS: central nervous system
ENS: embryonic neural stem
EPM: elevated plus maze
SGZ: subgranular zone
TBI: traumatic brain injury
